# Talking about Familial Breast and Ovarian Cancer Risk—Evaluation of a Psychosocial Training Module for Gynecologists in Germany

**DOI:** 10.3390/cancers16020310

**Published:** 2024-01-11

**Authors:** Friederike Kendel, Dorothee Speiser, Karen Fechner, Christine Olbrich, Stephanie Stegen, Alina Rörig, Markus A. Feufel, Stephanie Haering

**Affiliations:** 1 Gender in Medicine, Charité—Universitätsmedizin Berlin, Corporate Member of Freie Universität Berlin, Humboldt—Universität zu Berlin, and Berlin Institute of Health, Charitéplatz 1, 10117 Berlin, Germany; stephanie.haering@charite.de; 2 Hereditary Breast and Ovarian Cancer Center, Charité—Universitätsmedizin Berlin, Corporate Member of Freie Universität Berlin, Humboldt—Universität zu Berlin, and Berlin Institute of Health, Charitéplatz 1, 10117 Berlin, Germany; dorothee.speiser@charite.de (D.S.); karen.fechner@charite.de (K.F.); christine.olbrich@charite.de (C.O.); stephanie.stegen@charite.de (S.S.); 3 BRCA-Netzwerk e.V., Thomas-Mann-Str. 40, 53111 Bonn, Germany; 4 Division of Ergonomics, Department of Psychology and Ergonomics (IPA), Technische Universität Berlin, Marchstr. 23, 10587 Berlin, Germany; alina.roerig@tu-berlin.de (A.R.); markus.feufel@tu-berlin.de (M.A.F.)

**Keywords:** familial breast and ovarian cancer risk, primary care, genetic counseling, communication, training, medical education

## Abstract

**Simple Summary:**

Primary care gynecologists play an important role in setting the course for coping with an increased genetic cancer risk. The suggested 90 min psychosocial training module enables primary care gynecologists to expand their competence in dealing with familial breast and ovarian cancer burden. It may contribute to a sustainable improvement in the care of women with an increased risk of familial breast and ovarian cancer.

**Abstract:**

Primary care gynecologists are increasingly integrated into the care of patients with hereditary breast and ovarian cancer (HBOC) risks. These physicians should not only have basic genetic knowledge; they should also feel able to sensitively address an increased HBOC risk and deal with emotional, stressful situations in this context. Our project aimed at developing a training module, ‘iKNOWgynetics’, addressing psychosocial challenges in the context of HBOC care for primary care gynecologists. We developed the psychosocial training module in three phases: first, we conducted an online survey with *n* = 35 women with a family history of breast or ovarian cancer to assess patients’ experiences and needs. Second, based on the results of the needs assessment, we developed the training module. Third, we evaluated the training by assessing physicians’ (*n* = 109) self-efficacy with regard to communication skills in the context of HBOC before and after the training. In the needs assessment, seven psychosocial themes emerged. These themes, complementing a review of the literature, informed the training curriculum. The training was divided into two parts: (1) communicating with women before genetic testing and (2) care co-management for women with HBOC after genetic testing. After the training, participants reported a significant increase in self-efficacy in three domains: communicating empathetically, educating patients in a comprehensible way and dealing with emotionally challenging situations. Our results highlight the relevance of psychosocial issues for patients with HBOC. A genetic literacy training module that integrates aspects of psychosocial care increases physicians’ confidence in dealing with emotionally challenging situations before and after their patients’ genetic testing. Thus, such trainings may improve the care of women with hereditary cancer risks.

## 1. Introduction

Knowledge about hereditary cancers has increased rapidly in recent years, and genetic testing has become widely accessible [1,2]. Because knowledge of a pathogenic germline variant in *BRCA1/2* opens up new possibilities for early detection, prevention or therapeutic options, identifying a hereditary risk for breast and ovarian cancer at an early stage presents a great opportunity. Physicians in primary care can play a key role here. By taking a patient’s family history of cancer as part of their routine medical assessment, they could identify healthy women at risk even before they develop cancer [3,4,5].

However, talking about increased familial or genetic risk outside of specialized centers for genetic counseling is anything but easy. Information on genetic cancer risks is complex and rapidly changing, which may keep physicians from discussing these risks to avoid communicating outdated or incorrect knowledge [6]. A review including 48 studies showed that a further barrier is the concern that this topic may cause too much psychological distress for the patient [7]. Most health care professionals who are not specialized in genetics perceive their general communication skills with regard to HBOC as positive. Nevertheless, they have the impression that managing the emotionally stressful situations that arise due to this topic is difficult [8]. Another challenge is the co-treatment of women after genetic analyses. Especially in the first months after the detection of a pathogenic germline variant, women experience increased worry [9], and a large proportion of women significantly over- or underestimate their individual risk of actually developing breast or ovarian cancer [10]. A reasonable understanding of one’s individual cancer risk is, however, an important prerequisite for making sustainable decisions. Therefore, hereditary risks need to be conveyed sensitively [11,12].

Patients generally have high expectations of their physicians’ communication skills, and there is evidence that patient-centered communication is associated with higher patient satisfaction, increased understanding of information and better health outcomes [13]. Contrary to wide-standing beliefs, a sensitive communication style is not a matter of talent but is a core skill that can be acquired with adequate training, just like with surgical techniques [14,15].

In parallel with more and more physicians being integrated into the care of patients with hereditary cancer risks [16], continuing education programs for this target group that combine technical medical knowledge and psychological content have been developed. For example, Fallowfield et al. (2022) developed a training program for health care professionals who are involved in communicating *BRCA1/2* genetic test results in the UK. They demonstrated that knowledge, communication skills and confidence improved significantly after the intervention [17]. Notably, genetic counselling practices and referral systems differ widely between countries [18]. Continuing genomics education thus needs to be targeted to the specialty and role of participants [19] as well as country-specific legislation. In Germany, a targeted training program does not exist yet. Therefore, our multidisciplinary project aimed at developing the training ‘iKNOWgynetics’ to increase genetic literacy and communication skills among physicians who are not specialized in genetics, who may refer women at risk for HBOC to specialized centers for genetic counseling or who co-manage patients after the detection of pathogenic germline variants. The training consists of several modules that integrate basic genetic knowledge [10] as well as psychosocial content. Our didactic concept is based on the experience that both professional competence and self-efficacy are necessary for the successful transfer of educational programs into a clinical setting and that programs are especially successful when knowledge dissemination is connected with personal awareness and its application [17,20,21,22]. In this article, we focus on the module with psychosocial content. With this module, we aim to create awareness for the psychosocial needs associated with HBOC and to empower physicians who are not specialized in genetics to address these topics and successfully deal with emotionally challenging situations. We assumed that the training would lead to an increase in participants’ self-efficacy regarding patient-centered communication about HBOC.

## 2. Materials and Methods

The project was conducted in a 3-step procedure: assessment of patient needs (Phase 1), development of the training (Phase 2) and evaluation (Phase 3). For each phase, we present our respective procedure and results separately, and then discuss the results all together. With this approach, we aimed to increase transparency and comprehensibility.

### 2.1. Phase 1: Needs Assessment

To better understand patients’ experiences and expectations regarding discussions of family cancer burden with their primary care gynecologist, we conducted an online survey among women with a family history of breast or ovarian cancer and/or a pathogenic germline variant. We used a modified version of the German Measure of Patients’ Preferences (MPP-D) [23] to assess patients’ experiences with regard to eight communication characteristics, with each on a 4-point Likert Scale. We also assessed whether gynecologists had taken a family history of cancer—and, if so, regularly or only once. Open-ended text fields were added for an in-depth understanding of patients’ experiences and expectations (e.g., ‘What went well during the discussion of family cancer burden with your primary care gynecologist?’; ‘What do you expect from a primary care gynecologist with regard to familial breast and ovarian cancer?’) and analyzed answers with qualitative content analysis [24]. The study was approved by the medical school’s data protection authorities and ethics committee (EA4/035/20; 27 February 2020).

#### Results from the Pilot Study on Patients’ Experiences and Needs

Thirty-five women completed the online survey. Mean age was 48.1 ± 10.2 years, with 82.8% of women reporting a high school degree. Except for two participants, all women reported a family history of cancer and 62.9% had received a breast or/and ovarian cancer diagnosis before. A total of 42.9% of the women were first informed about the possibility of genetic testing by their oncologist, 22.9% by their relatives and 17.1% by their gynecologist (see Supplement, Table S1).

**Figure 1:**
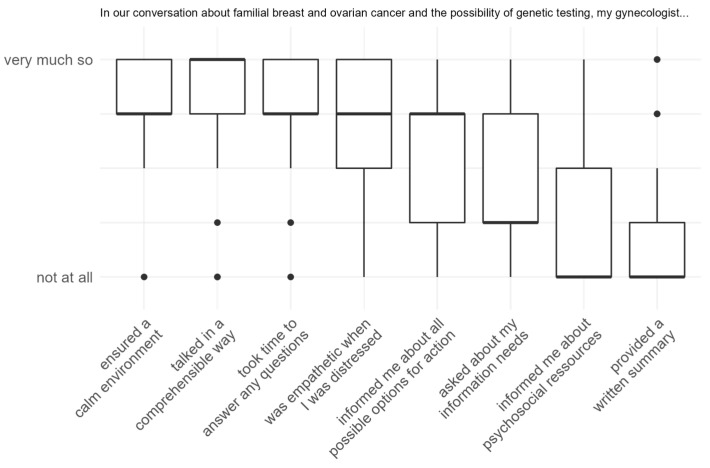
Boxplots of MPP-D items measuring patients’ experiences with their primary care gynecologist when discussing their family history of cancer.

While 77.1% of patients indicated that their primary care gynecologist had asked about cancer in the patient’s family during the initial visit, only 25.7% stated that they were asked about changes in their family cancer history at regular intervals. In total, 71.4% of women had discussed HBOC issues with their primary care gynecologist, and nearly all of them (88%) rated the conversation as ‘good’ or ‘rather good’. Figure 1 depicts the distribution of eight conversation characteristics: Patients reported that their physician ensured a calm environment (M = 3.28 ± 0.94), talked in a comprehensible way (M = 3.16 ± 1.28) and took time to answer questions (M = 3.04 ± 1.02) during their consultation. They indicated a moderate to high level of empathy during distressing situations (M = 3.00 ± 1.08) and medium levels of receiving information about all possible options for action (M = 2.36 ± 1.38) and being asked about their information needs (M = 1.72 ± 1.31). The amount of information about psychosocial resources was rated low (M = 1.00 ± 1.23), as was the probability of obtaining a written summary of the information discussed (M = 0.96 ± 1.31).

Qualitative analysis of patients’ experiences and expectations highlighted the need for integrated care: both professional expertise as well as psychosocial aspects of care were reoccurring themes. Among psychosocial aspects, the following sub-themes emerged: patients indicated that they value when their physician (1) provides emotional support, (2) adopts a sensitive communication style and (3) shows genuine interest in their patient. Moreover, patients expressed (4) their wish to be taken seriously with their concerns, and (5) to have enough time to discuss these concerns with their physicians. They also highlighted that they would have liked their physician to point them towards (6) trustworthy information materials and (7) psychosocial resources such as peer support groups.

Pars pro toto, we quote a patient: ‘It is certainly not easy for a gynecologist to find the right balance between thorough examination (…) and avoiding to alarm a patient unnecessarily. In addition, there is the time pressure during a consultation. Together, this can lead to a situation where a patient may feel overly demanding or overly concerned if she brings up the possibility of a genetic predisposition. (…) It would be desirable to allow a serious discussion of the subject.’

### 2.2. Phase 2: Development of the Training Curriculum

We consolidated information from multiple resources to develop our training curriculum: (1) We conducted a scoping literature review on best practices for communicating emotionally stressful information. (2) We also drew upon our previous work on risk communication [12], (3) the results of the needs assessment, (4) a former assessment of physicians’ needs (see Speiser et al., [10]) and (5) professional knowledge and clinical experience from our multidisciplinary team, including clinicians, psychologists, human factor and ergonomics specialists, and expert patients to refine the content in an iterative process. The psychosocial module was integrated into a live online-seminar of the iKNOWgynetics training (for more details, see [10]).

#### 2.2.1. Description of the Training Intervention

The interactive 90 min psychosocial module covers the unique situation of women with familial breast or ovarian cancer burden and is divided into two parts: First, we discuss psychological aspects of the special situation of women before referral to genetic counseling and testing. In the second part, we address issues that are important when co-counseling women who already have received a positive genetic test result. Table 1 provides an overview of the content, learning objectives and duration of the respective course units.

#### 2.2.2. The Situation before Genetic Testing—Part A

In the first part of the module, we focus on the special situation of women with HBOC before genetic testing. Using examples from the patient survey, we address patients’ psychosocial expectations and highlight the need for better integration of psychosocial aspects into the care of women with HBOC burden. To create awareness for the specific needs and potential strains at this stage, we discuss inclusion criteria for hereditary breast and ovarian cancer burden through a psychosocial lens. It is emphasized that certain factors, such as a young age of cancer onset and the accumulation of cancer in close family members, which present biomedical criteria for an increased risk, may also confer a particular psychological burden.

**Figure 2:**
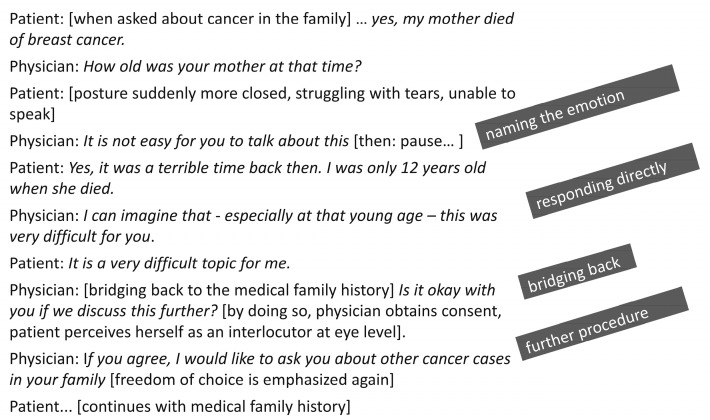
Example of patient-oriented communication. How can the physician deal with emotions without letting go of the goal of the conversation?

We convey the principles of patient-oriented communication using various case studies. The first three case examples demonstrate different challenging situations: (a) a patient who meets indication for testing and is very concerned; (b) a patient who also meets the criteria, but strongly opposes testing; and (c) a patient who does not meet criteria for testing but is seriously worried and still requests testing. In all the examples, we practice basic principles of communication: how to verbalize emotions, responding to what was said and how to build a bridge back to the actual topic of conversation. One example is given in Figure 2.

To illustrate different communication styles for physicians and their potential impact on patient interaction, we created three videos of the same situation with different communication styles in each: Following Schmid Mast et al. [25], one scenario is emotion-centered, i.e., the physician over-emphasizes the patient’s emotions, and takes breaks which are too long and difficult to endure. Another scenario is physician-centered, i.e., the doctor talks proportionally more than the patient, conveys too much information, uses many medical terms and hardly takes any time for pauses. The third scenario is patient-centered and serves as a positive example of a balanced doctor–patient interaction that values the patient’s perspective. Finally, the SPIKES model [26], which was originally developed for the context of breaking bad news, is used to integrate patient-centered communication when addressing genetic testing into a larger theoretical framework. The SPIKES model comprises the components ‘Setting of the interaction’, ‘Patient’s perception and preparation’, ‘Invitation and information need’, ‘Knowledge of the information’, ‘Empathy and exploration’ and ‘Summary and strategic planning’ [26]. We encourage participants to use the SPIKES model as a checklist, particularly to analyze and reflect on difficult conversations.

#### 2.2.3. Relevant Aspects When Discussing the Topic ‘Familial Cancer Burden’ with Women after Genetic Testing—Part B

The second part of the training module revolves around issues that are particularly relevant for women after genetic testing. In the beginning, we present results from a recent study on patients’ understanding of their individual risk for breast and ovarian cancer [12] and interactively discuss possible reasons for over- and underestimation, such as a lack of numerical understanding, previous experiences, emotional stress or the perceived lack of controllability. Against this background, participants are trained on how to address these aspects to improve risk understanding. In line with patients’ expectations, expressed in the needs assessment, special attention is given to the role of peer networks and access to evidence-based information. We particularly highlight evidence for the effects of lifestyle changes on breast cancer development and progression [27] and the contribution of a healthy lifestyle to quality of life [28].

### 2.3. Phase 3: Evaluation of the Training Curriculum

#### Methods of Evaluation of the Training

Based on sampling experiences from similar studies [17,29], our study aimed to recruit at least *n* = 100 participants. To evaluate the training, we assessed participants’ self-efficacy expectations regarding patient-centered communication in the context of genetic testing. Following the classic example of Schwarzer and Jerusalem [30], we adapted three items on self-efficacy expectation using a 4-point scale from 0 (not at all true) to 4 (exactly true). T-tests for dependent samples were used to test changes in self-efficacy expectations before and after the training. Cohen’s d was calculated as effect size. All analyses were performed in statistical environment R version 4.0.3 [29]. The study was approved by the institutional ethics committee (AR_01_20200608; 8 July 2020).

## 3. Main Results—Evaluating the Training

One hundred and nine physicians attended one of five workshops between August 2020 and February 2021. Complete pre- and post-workshop data were available for 103 participants (89.3% women, mean age = 50.75 ± 9.02 years), of whom 82.5% were gynecologists in ambulant care, 12.6% were gynecologists in a hospital and 4.85% indicated another specialty (e.g., oncology, radiology or research). The average professional experience was M = 24.05 ± 8.46 years. The majority (93%) of the gynecologists worked in an urban environment. Of all participants, 56.5% worked in a solo practice, whereas 43.5% were part of a group practice (for more detail, see Speiser et al. [10]).

Improvements in attendees’ self-efficacy expectations across all three aspects evaluated were highly significant (see Table 2): communicating in an empathetic way, educating patients in a comprehensible way and feeling confident in dealing with emotionally challenging situations. Cohen’s d for correlated measurements indicated small to medium effect sizes.

## 4. Discussion

Our results show that a curriculum combining complex genetic topics with a focus on patient-centered communication strategies significantly increases physicians’ self-efficacy regarding patient interactions. After the training, physicians felt more confident than before the training to discuss an increased risk for a genetic mutation with their patients, to communicate medical content to patients in an understandable way and, most importantly, to cope with challenging emotional situations in this context. The simultaneous teaching of complex technical knowledge and communication skills, which has also been successful in other trainings [20], presents a valuable expansion of biomedical education alone.

Our training, which focused on primary care gynecologists in Germany, is another component on the path toward more comprehensive care for women with a family history of cancer. It expands previous international efforts on this endeavor, such as coaching for specialized nurses to facilitate shared decision making for healthy *BRCA1/2* gene mutation carriers [31]; a training program for oncologists, nurses and genetic counselors [17]; and an online training program for medical specialists in cancer genetics, which was recently evaluated in a pilot study [32]. Our training is tailored to the German health care context and country-specific legislation with regard to genetic testing, while on the other hand integrating international findings on evidence-based care.

Primary care gynecologists who are not specialized in genetics, and for whom we designed the training, play a major role in identifying familial cancer risk. Often, gynecologists care for female family members across generations and therefore have an overview of the individual’s family cancer burden. Overall, the willingness of women to undergo genetic testing following a targeted recommendation has increased in recent years [2]. Nevertheless, a substantial number of women with a familial burden do not undergo genetic testing before cancer is diagnosed [33,34]. The results of our needs assessment align with this finding. Less than one-fifth of women reported that they had been told about the possibility of genetic testing by their primary care gynecologist. Hence, primary care gynecologists may raise awareness and provide information to healthy women who particularly benefit from early preventive measures. Because the procedure of genetic testing may cause a great psychological burden for the women [35], it is important to deal with this topic sensitively and empathically from the very beginning. Bonadona et al. [36] emphasized that the way experts deal with their patients’ concerns may be crucial to how women cope with the procedure and how they themselves pass the information on to their relatives. In particular, women who received extensive counseling prior to genetic testing reported having less distress in the long term [37].

During their medical education, physicians learn the basics of patient-centered communication and how to deal with psychological distress in patients. This knowledge must be continuously reflected and transferred to specific areas. For one thing, familial cancer is a very sensitive issue and women bring their very own experiences due to their family history: many women have witnessed their sister, mother, aunt or cousin develop cancer and, in the worst case, die of it. An empathic and encouraging approach, with room for fear and other emotions, is most likely to help reduce anxiety [38] and is a prerequisite to calmly discuss appropriate medical measures. Another challenge in everyday practice is the great time pressure. Consultation time per patient is limited, which usually cannot be changed. This makes it even more important to create a good conversation within limited time. Here, the perception and verbalization of emotions is an important tool for good and mutually satisfying communication. The results of our needs assessment further show that women have a great need for additional information and resources. Therefore, the discussion of lifestyle changes (diet, physical activity, stress management and support groups) is part of the training.

The training and its evaluation have several limitations. The majority of women participating in the patient needs assessment had obtained high school education. As women with lower educational statuses may be less likely to have their increased cancer risk addressed [39] or may experience more problems with incomprehensible language compared to women with higher education levels, our results may not be generalizable to patients from lower socio-economic backgrounds. More detailed examinations on health disparities and diverse needs in genetic counseling and testing are necessary to serve women from all backgrounds equally. Moreover, a more nuanced understanding of patient experiences by diagnostic status (i.e., patients with familial history of gynecological cancer but whose own risk status is unknown, patients with a mutation but who are currently not diagnosed with cancer, patients with a mutation and diagnosed cancer) might help us to develop tailored interventions that address the specific needs of that target group. For the physician training, we switched to an online format due to the pandemic—which changed the interaction mode with the participants. With careful planning and active encouragement of participants’ exchange, we aimed to counteract the lack of direct interactions in the virtual format. One clear advantage of the online format was the time saved: many participants reported that they would not have joined an offline training that required additional time to get to the venue and back. Third, post-training evaluation was only conducted shortly after the training. Thus, we cannot make inferences about long-term effects in self-efficacy changes. Future research should therefore examine the sustainability of the observed changes. In addition, the benefit of the training could be investigated in a randomized controlled trial that includes a non-intervention control group as well as patient-reported outcome measures.

One important lesson learned was that many physicians tend to focus on acquiring (bio-) medical content. To increase interest in psychosocial issues, the psychosocial content should therefore be linked to clinical examples as much as possible and include the experiences of the participants.

## 5. Conclusions

Primary care gynecologists play an important role in setting the course for coping with an increased genetic cancer risk. As the first multidisciplinary training of this kind in Germany, the 90 min psychosocial training module of iKNOWgynetics enables primary care gynecologists to expand their competence in dealing with familial breast and ovarian cancer burden. The combination of medical content and communication skills increases physicians’ self-efficacy expectations to better meet patients’ psychosocial needs and to deal with challenging situations in the context of genetic testing. The extent to which this training positively influences the identification and care of women with a family history of cancer risk should be examined in further intervention studies.

## Figures and Tables

**Table 1:** Training curriculum: situation before and after genetic testing. Content, learning objectives and duration.

Content	Learning Objective Participants Should…	Duration (Minutes)
Part A: the situation before genetic testing (45 min)		
Psychosocial expectations and experiences of women with their gynecologists	… become aware of specific needs	5
Inclusion criteria for familial breast and ovarian cancer risk from a psychosocial perspective	… become aware of potential strains	5
Case examples	… be able to apply basic principles of communication in emotionally challenging situations	25
SPIKES model	… be able to use the SPIKES model to reflect on difficult situations	10
Part B: the situation after genetic testing (45 min)		
Presentation of data on under- and overestimation of cancer risk in women with HBOC	… be able to recognize and address over- and underestimation	25
Peer networks, access to information	… become aware of the potential role of peer networks	10
Lifestyle changes	… be able to address the importance of lifestyle changes for health-related quality of life	10

**Table 2:** Changes in self-efficacy expectations regarding patient-centered communication in the context of genetic testing.

	Pre-Workshop M (SD)	Post-Workshop M (SD)	Cohen’s d	t (df)	*p*-Value
I know how to communicate an increased risk for BRCA mutation in an empathic way	3.16 (0.60)	3.32 (0.53)	0.22	2.31 (102)	0.012
It is easy for me to educate my patients about familial cancer in a comprehensible way.	2.94 (0.65)	3.28 (0.47)	0.49	4.97 (102)	<0.001
I am confident in dealing with different emotionally challenging patient reactions	3.00 (0.54)	3.20 (0.45)	0.37	3.77 (102)	<0.001

## Data Availability

The data that support the findings of the study are available from the corresponding author upon reasonable request.

## Supplementary Materials

The following supporting information can be downloaded at: https://www.mdpi.com/article/10.3390/cancers16020310/s1, Table S1: Participant characteristics (*n* = 35).
