# Supplementary material for: Talking about Familial Breast and Ovarian Cancer Risk—Evaluation of a Psychosocial Training Module for Gynecologists in Germany
Source: Cancers (Basel). 2024 Jan 11;16(2):310. doi: 10.3390/cancers16020310 (PMC10813984; doi:10.3390/cancers16020310)
Supplement: Supplementary file 1 [file cancers-16-00310-s001.zip › cancers-2792199-supplementary.pdf]

Supplementary Table S1. Participant characteristics (*n* = 35).

Female sex, n (%)	35 (100)
Age, M (SD)	48.1 (10.2)
Higher education[1], n (%)	29 (82.8)
Diagnosis of breast cancer, n (%)	19 (54.3)
Diagnosis of ovarian cancer, n (%)	6 (17.1)
No diagnosis of cancer, n (%)	13 (37.1)
Number of breast or ovarian cancer diagnoses in the family, n (%)	
0	2 (5.7)
1	11 (31.4)
2	8 (22.9)
3 or more	14 (40.0)
First information about genetic testing was obtained by/through…	
oncologist, n (%)	15 (42.9)
family/relatives, n (%)	8 (22.9)
gynecologist, n (%)	6 (17.1)
friends, n (%)	2 (5.71)
internet, n (%)	1 (2.86)
other, n (%)	3 (8.57)

Notes. [1]Higher education refers to ≥12 or 13 years of schooling (highest educational degree in the German schooling system).
